# Genotyping *Porcine Circovirus 3* (PCV-3) Nowadays: Does It Make Sense?

**DOI:** 10.3390/v12030265

**Published:** 2020-02-28

**Authors:** Giovanni Franzo, Eric Delwart, Robert Fux, Ben Hause, Shuo Su, JiYong Zhou, Joaquim Segalés

**Affiliations:** 1 Department of Animal Medicine, Production and Health (MAPS), Padua University, 35020 Legnaro, Italy; 2 Vitalant Research Institute, San Francisco, CA 94118, USA; Eric.Delwart@ucsf.edu; 3 Department of Laboratory Medicine, University of California, San Francisco, CA 94118, USA; 4 Institute for Infectious Diseases and Zoonoses, Department for Veterinary Sciences, LMU Munich, 80539 Munich, Germany; robert.fux@micro.vetmed.uni-muenchen.de; 5 Kansas State Veterinary Diagnostic Laboratory, Kansas State University, Manhattan, KS 66506, USA; hausebm@gmail.com; 6 MOE Joint International Research Laboratory of Animal Health and Food Safety, Jiangsu Engineering Laboratory of Animal Immunology, Institute of Immunology and College of Veterinary Medicine, Nanjing Agricultural University, Nanjing 210095, China; ssh5658485@163.com; 7 MOA Key Laboratory of Animal Virology and Department of Veterinary Medicine, Zhejiang University, Hanghou 310058, China; jyzhou@zju.edu.cn; 8 Departament de Sanitat i Anatomia Animals, Facultat de Veterinària, UAB, 08193 Bellaterra, Spain; joaquim.segales@irta.cat; 9 UAB, Centre de Recerca en Sanitat Animal (CReSA, IRTA-UAB), Campus de la Universitat Autònoma de Barcelona, 08193 Bellaterra, Spain; 10 OIE Collaborating Centre for the Research and Control of Emerging and Re-emerging Swine Diseases in Europe (IRTA-CReSA), 08193 Bellaterra, Barcelona, Spain

**Keywords:** PCV-3, genotypes, classification, ORF2, genome

## Abstract

The discovery of a globally distributed porcine circovirus (*Porcine circovirus 3*; PCV-3) has led to intense research activity and the production of a large amount of molecular data. Different research groups have proposed several, not always concordant, genotypes for this virus. While such categories could aid an easier interpretation of PCV-3 molecular epidemiology, any classification, to be useful in practical settings, must be univocal and of help in the understanding of underlying biological features and epidemiology. Based on these premises, the possibility of defining PCV-3 genotypes was evaluated on the broadest available dataset of PCV-3 complete genome (*n* = 357) and open reading frame 2 (ORF2, *n* = 653) sequences. Genetic distance and phylogenetic clustering were selected as the main objective criteria. Additional factors, including the number of within-cluster sequences, host and geographic clustering, concordance between different genomic regions, and analysis method were also taken in account to generate a classification that could be effectively applied in research and diagnostic settings. A maximum within-genotype genetic distance of 3% at the complete genome and 6% at the ORF2 levels, bootstrap support higher than 90%, and concordance between analysis methods allowed us to clearly define two clades which could be potentially defined as genotypes. Further subdivision was not suggested due to the absence of a meaningful association between PCV-3 and its biological/epidemiological features. Nevertheless, since one of the clades included two strains only, thus far we formally propose the definition of only one PCV-3 genotype (PCV-3a). The established criteria will allow us to automatically recognize other genotypes when more strain sequences are characterized.

## 1. Introduction

Porcine circovirus 3 (PCV-3) is a recently identified member of the genus *Circovirus.* It is featured by a single-stranded circular DNA genome of 1999–2001 nt. Similar to other circoviruses, two main open reading frames (ORFs) have been identified. ORF1 is considered the most conserved region of the circovirus genomes. It is located on the positive strand and codes for a single replicase protein (Rep) of 296–297 aa for PCV-3 [1,2,3]. ORF2 is located on the negative strand and encodes the Cap protein (about 214 aa), the only constituent of the viral capsid, which is considered the most variable and most immunogenic viral protein [1]. Nevertheless, it must be stressed that current knowledge on PCV-3 immunology is very limited and essentially based on comparison with porcine circovirus 2 (PCV-2) and/or on in silico evolutionary studies [4]. Another putative ORF (ORF3) would code for a putative 231-aa protein, whose function has not been elucidated yet; it has been predicted to be oriented in the opposite direction of ORF1 [2].

Since its first identification in the United States thorough a metagenomics approach [2,3], a remarkable amount of epidemiological and molecular data has been accumulated regarding PCV-3. This virus has been reported in all continents (with Africa and Australia being the only exceptions so far) at moderate to high rates of detection (>10%), depending on the particular study, country, and farm (reviewed in Klaumann et al., 2018 [1]). At the same time, PCV-3 DNA has been found in samples from pigs with several clinical syndromes, including respiratory, reproductive, gastrointestinal, and neurological disorders [2,3,5,6]. Nevertheless, the virus has also been detected in healthy animals [7]. Therefore, at the present state, although there are strong suspicions of associations with reproductive disease and multisystemic inflammation [8], the causative role of PCV-3 in the observed clinical conditions has not been confidently defined [9]. In addition, the circulation of strains with different virulence has not been reported.

Besides domestic pigs, PCV-3 has been detected in other domestic species, including dogs [10,11], cattle [12], and mice [13], and wild species, including wild boars [14,15,16], chamois, roe deer, and associated ticks [15]. However, only wild boar seems to be infected at high prevalence and might play a significant role in PCV-3 epidemiology. 

The identification of a potential association between biological and epidemiological features (e.g., virulence, geographic distribution, and host tropism) with genetic features is of great interest. The willingness to provide a simplified depiction and interpretation of PCV-3 genetic features has led to the definition of different genetic groups, typically named PCV-3a, PCV-3b, and PCV-3c, or Groups A and B [17,18,19]. Similar to what initially occurred for PCV-2 [20], no consensus is present and different research groups have proposed independent classification criteria and schemes, leading to a certain confusion among veterinarian and researchers. In order to be practical and useful, the PCV-3 classification must not be for its own sake, but should be of help in the interpretation of the underlying viral features and, thus, in the understanding of its epidemiology and potential control measures [21].

Based on these premises, the applicability of a PCV-3 genotyping proposal is evaluated in the present work, offering standardized criteria based on a consensus shared by different research groups operating around the world. 

## 2. Material and Methods

### 2.1. Database Preparation

All available PCV-3 complete genome sequences were downloaded from Genbank (Accessed 19/10/2019).

Similarly, all ORF2 sequences were downloaded and only complete ones were maintained for further analysis. When available, collection year, country, and host were extracted from the record and associated to the sequence.

Complete genome sequences were aligned using MAFFT [22], while Cap-encoding ones were translated at amino acid level, aligned using MAFFT, and then back-translated to nucleotides using TranslatorX [23]. All poorly aligned sequences and those reporting ambiguous nucleotides, frame-shift mutations, and premature stop codons were removed from the alignment. 

Potential recombinant strains were identified using RDP4 [24] and removed from the alignment. The RDP4 settings for each method were adjusted to account for the dataset features according to the RDP manual recommendations. Only recombination events detected by more than one method with a significance value lower than 0.01 (*p*-value < 0.01) and Bonferroni correction were accepted.

The presence of undetected recombination breakpoints was evaluated using the SBP algorithm implemented in HYPHY [25].

To facilitate the analysis, reduce the computational burden, and increase tree interpretability, only unique sequences were maintained in the alignment. Therefore, only one sequence was selected as representative of all identical ones.

### 2.2. Phylogenetic and Cluster Analyses

The relationship between PCV-3 strains was evaluated by phylogenetic analyses.

Three different tree reconstruction methods were used: neighbour joining (NJ) using MEGA X [26], maximum likelihood (ML) using RaxML [27], and Bayesian inference (BI), using MrBayes [28]. The best substitution method was selected for both complete genomes and ORF2 sequences based on the Bayesian Information Criteria (BIC) calculated using Jmodeltest [29].

The robustness of clusters identified by NJ and ML was assessed performing 10,000 bootstrap replicates. For the Bayesian analysis, two independent runs, including one cold and three heated MCMCMC chains, were run for 10,000,000 generations, sampling model parameters and trees every 5000 generations. The runs convergence and mixing were visually inspected and results accepted only if estimated sample size (ESS) was higher than 200 and the potential scale reduction factor (PSRF) approached 1. A consensus tree was obtained after discarding the first 25% of estimated trees as burn-in. The posterior probability of each clade, estimated calculating the proportion of the time that each bipartition was found in the posterior trees, was used as a measure of clade robustness.

The presence of reliable clusters that could be considered as genotypes was evaluated according to two criteria: raw genetic distance and bootstrap/posterior probability support. Clusters were identified and marked using ClusterPicker [30] by evaluating different combinations of raw genetic distances (i.e., maximum within-cluster genetic distance) and bootstrap support. 

### 2.3. Association between PCV-3 Phylogeny and Epidemiological Features

To assess the association between qualitative traits (i.e., geographical location and host) accounting for phylogenetic uncertainness, different statistics (parsimony score (PS), association index (AI) and monophyletic clade size (MC) statistics) were calculated using BaTS [31]. A Bayesian analysis was performed on the whole ORF2 dataset. Since a limited number of complete ORF2 sequences were available for hosts other than domestic swine, a partial ORF2 dataset was also created and analysed. The considered region was arbitrarily selected in order to achieve the best compromise between the sequence length and the number of included sequences. The posterior trees were used for BaTS analysis after discarding the first 20% of the trees as burn-in. 

The abovementioned statistics were calculated across all these trees and the observed median (μ obs) value was selected as the final outcome. The distribution under the null hypothesis of no trait–phylogenesis association was obtained by randomizing the tip–trait association 1000 times without replacement for each tree of the posterior distribution. For each randomized dataset the statistics medians (μ null) were calculated and formed the null distribution. This distribution was used to achieve a p-value by simply evaluating the proportion of simulated values more extreme than the observed one. The significance level was set to *p* < 0.01.

## 3. Results

After database refinement, 357 complete genomes and 653 complete ORF2 sequences were included in the study. The geographic and host distribution of the included sequences is reported in Table 1.

**Figure 1:**
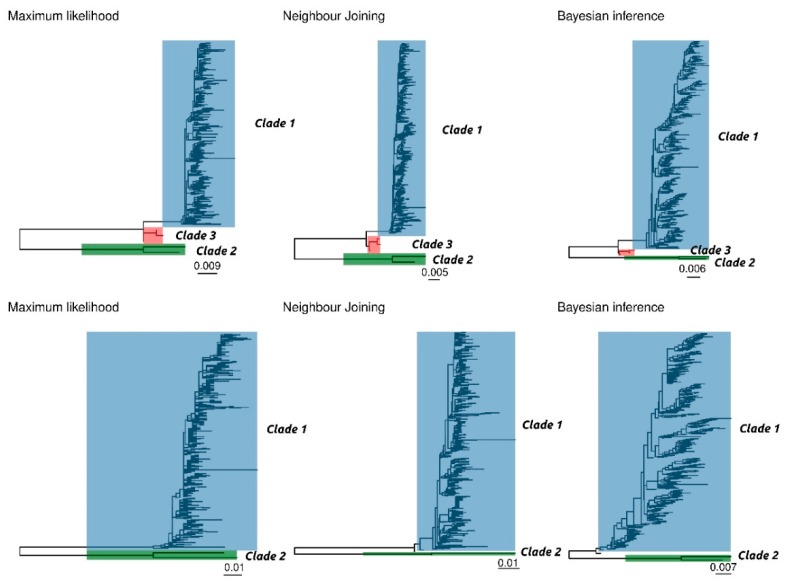
Phylogenetic trees obtained on complete genome (**upper figure**) and ORF 2 (**lower figure**) using the maximum likelihood (ML), neighbour joining (NJ), and Bayesian inference (BI) approach. Different clades have been colour coded.

The results of ML, NJ, and BI of complete and ORF2 sequences provided overall concordant topologies, featured by a homogeneous genetic group and two distantly related sequences, corresponding to strains sampled in China in 2006 (Figure 1).

Several potential genetic distance thresholds and bootstrap values were evaluated in order to define a reasonable number of robust genotypes. Using a combination of 90% bootstrap support (or posterior probability) and a 3% and 6% within-clade maximum genetic distance for the complete genome and ORF2, respectively, two clusters were unequivocally identified, which included the same sequences independently of the selected method and dataset (Figure 1). The first one (Clade 1) comprises the vast majority of sequences, and it is featured by highly homogeneous sequences (complete genome average p-distance = 0.009, range = 0.000–0,026; ORF2 average p-distance = 0.014, range = 0.000–0,024). The second identified cluster (Clade 2) includes only the two above mentioned Chinese sequences (complete genome p-distance = 0.023, ORF2 p-distance = 0.057). The two clades are separated by a minimum p-distance of 0.078 and 0.121 at the complete genome and ORF2 level, respectively.

In the complete genome dataset only, another intermediate cluster (Clade 3) was identified using the above mentioned criteria. However, these Clade 3 sequences (GenBank accession numbers KY924473, KY924474 and KY924475) were part of Clade 1 in the ORF2 based analysis. Finally, in the ORF2 dataset, sequences MF318450 and MG253680 were not classified within any cluster using the NJ and BI methods, respectively. 

While additional sub-clusters could be identified within Clade 1, this was featured by a typically low bootstrap support (i.e., lower than 70%) (Supplementary Figure S1) and were not consistent among different dataset and analysis methods. 

A significant association was detected between tree topology and continent or host (Table 2). However, when a reduced dataset was prepared by down-sampling the number of sequences collected in Asia to obtain a more balanced dataset, no association between continent and tree topology could be demonstrated for most geographical regions (Table 2). Even when such association could be detected, it involved regions (i.e., Russia and South America) for which a very limited number of sequences was available (Table 1). Similarly, when the number of sequences collected from *Sus scrofa* was down-sampled, a lower statistical association, although present, could be detected between host and tree topology (Table 2).

**Figure 2:**
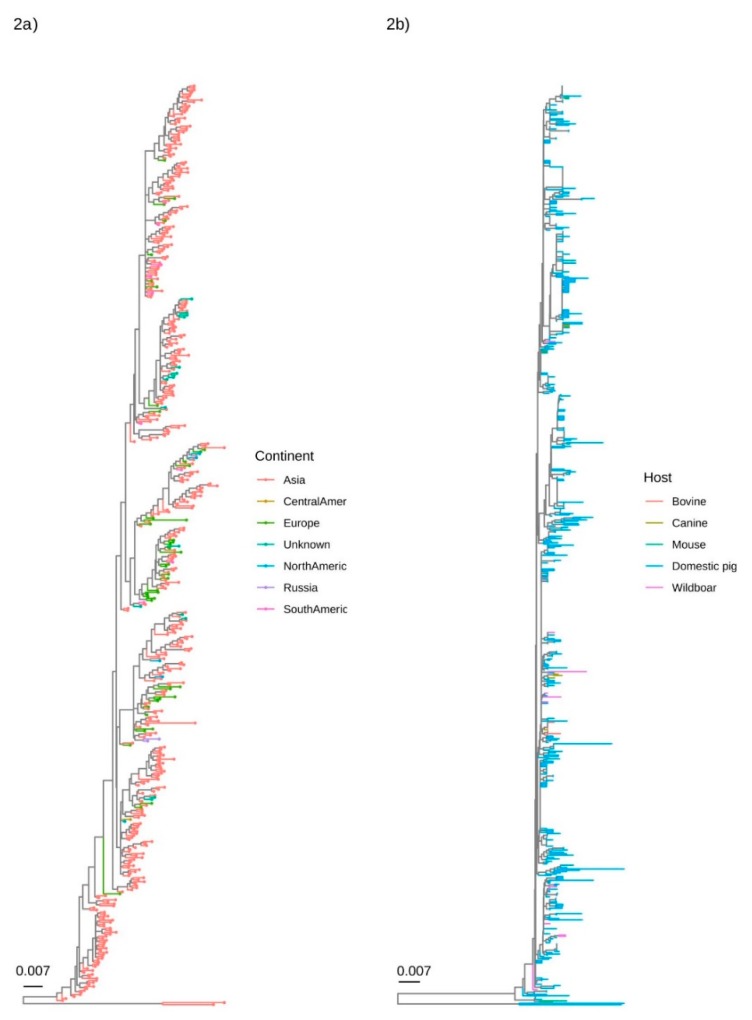
Phylogenetic tree reconstructed using the BI on the complete (**left figure**) and partial (**right figure**) ORF2 dataset. The collection continent and host have been colour-coded.

The qualitative evaluation of major clades demonstrated the absence of any geographical pattern. Although Asia was by far the most represented location, strains collected in other regions were well interspersed in the phylogenetic tree (Figure 2a). A similar scenario was demonstrated when the collection host was considered, with sequences obtained from different hosts mixed in different clades (Figure 2b).

**Figure 3:**
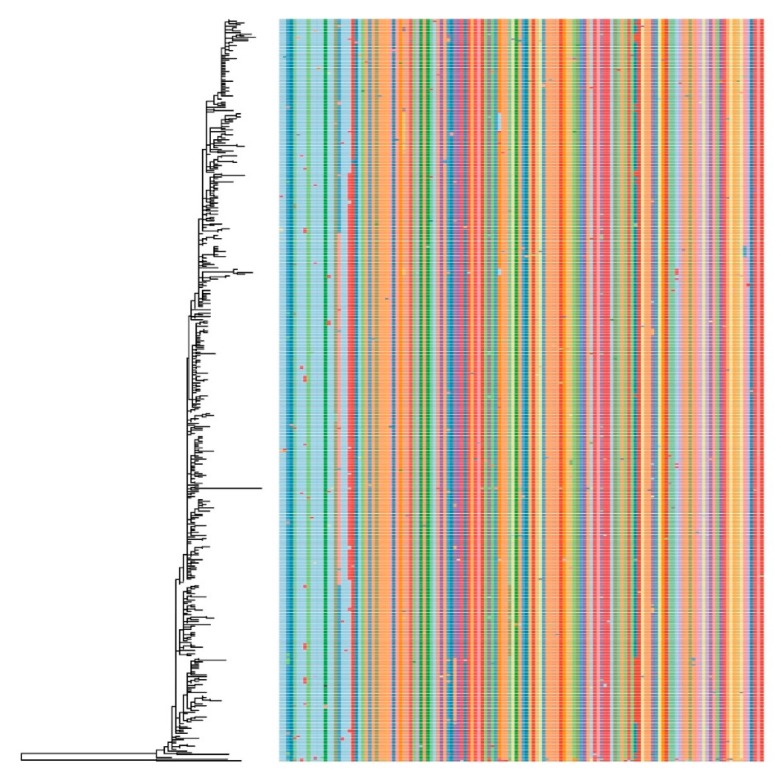
Plot reporting the alignment of the amino acids of the cap gene with respect to their position in the phylogenetic tree. Different amino acids have been colour-coded. For ease of interpretation, only the results of ML-based analysis are reported.

The presence of potential amino acid markers was evaluated on the ORF2 (Cap) dataset. Although a certain correspondence was observed between genotype and amino acid sequence, several exceptions were also present and no reliable amino acid markers differentiating PCV-3 in separate groups could be identified (Figure 3).

## 4. Discussion

The identification of a new porcine circovirus, likely because of its similarities with the more well-known and economically important PCV-2, has stimulated a remarkable number of studies on PCV-3, including the genetic characterization of several strains. To date, different research groups have introduced different nomenclatures in order to provide an easier-to-understand depiction of PCV-3 epidemiology [17,18,19,32]. Such classifications could represent a double-edged sword when no shared criteria are proposed and accepted at the international level. While the definition of categories can surely simplify and enhance the interpretation of PCV-3 epidemiology, the presence of different nomenclatures can lead to confusing and misleading result interpretations, as initially occurred for PCV-2 [33,34] and several other viral infections [35]. Additionally, the inclusion of strains in separate groups could erroneously lead to the conclusion that differences do exist even in biological features when those have not been demonstrated thus far. It must be taken into account that it is difficult to assess different biological features such as virulence, host adaptation, or tissue tropism for a virus that has not yet been isolated in cell culture. In consequence, reliable pathogenesis studies are lacking, although one experimental infection using a PCV-3 infectious clone has been published [36]. In addition, field data do not indicate that different PCV-3 strains cause different disease outcomes [1].

With this in mind, the broadest available dataset of complete PCV-3 ORF2 and genome sequences was analyzed to evaluate the feasibility and practical utility of establishing a shared PCV-3 classification.

Although the complete genome provided more informative sites, the whole genome sequencing is typically challenging, especially in routine diagnostic settings. Additionally, distance comparison over the entire virus genome of viruses that are subject to recombination, a phenomenon widely occurring in PCV-2 [37] and other circoviruses [38], is likely misleading [21]. ORF2 is the most variable PCV-3 genome region and likely the one most affected by host-derived immune pressure selection [1,4]. Its sequencing and analysis could therefore represent a reasonable cost/benefit compromise and was thus included in the analysis for more accurate evaluation.

Currently, no strict guidelines are present to define taxonomic levels under the species level by the International Committee for the Taxonomy of Viruses (www.ictvonline.com). Nevertheless, among the potential criteria, the present work attempted to adhere to the ones typically followed by official viral taxonomy, i.e., genetic distance and phylogenetic analyses [39,40]. Based on the selected criteria (bootstrap support > 0.9 and maximum genetic distance of 3% and 6% at the complete genome and ORF2 levels), two clades (Clade 1 and Clade 2) could be consistently identified independently from the considered region and analysis method. Particularly, Cluster 1 included strains which were previously divided in two or more genotypes (named PCV-3a, PCV-3b, and PCV-3c, or Groups A and B in previous papers) [17,18,19,32]. Even if the tree topology could support the definition of additional clades, the presence of several intermediate minor clades and the poor bootstrap support suggest caution in the recognition of these potentially different genotypes. Even though a certain geographic and host clustering could be statistically identified, this seems largely explained by the severely biased sequence availability, featured by a clear predominance of sequences collected from Asia (especially China) and *Sus scrofa.* Since most of the sequences collected from other hosts or continents (e.g., South and Central America, Russia, etc.) were obtained from single/few studies, an effect of the specific experiment rather than an actual difference in distribution is likely. This hypothesis is supported by the relevant reduction in statistical significance of the observed trait–genotype association when a more balanced dataset was artificially created. Future sequencing of strains collected from other hosts and non-Asian countries would be of help to further elucidate these issues. Nevertheless, when the most relevant genetic groups were evaluated, no clear association could be observed between specific clades and host or geographic distribution. These results confirm previous studies, which highlighted a broad PCV-3 distribution and a massive circulation involving countries all around the world [1,4]. 

Similarly, no evidence of a differential virulence has been reported to date. Setting a rational number of man-made categories onto a substantial continuum of viral evolution (i.e., genetic variability) requires pragmatism in order to ensure its utility and applicability by the virology community [21]. Therefore, the definition of genotypes should account not only for genetic features, but also for virus features that are designed to assist studies of its biology and/or epidemiology. Based on these considerations, no evolutionary, epidemiological, or biologic factors seem to justify the further division of Clade 1 in additional genotypes. This conclusion was also supported by the analysis of the amino acid profile. While initial studies suggested the presence of reliable amino acid markers [18], the updated dataset revealed that although a certain association between genotype and phenotype can be identified, several exceptions are present, likely due to random mutations or convergent evolution. 

Clade 2 included only two distantly related sequences collected in 2006 from two different Chinese farms. The farms experienced high mortality; however, they were also affected by a highly pathogenic porcine reproductive and respiratory syndrome. No further sequences of PCV-3 were found in the same farms (Dr. Jue Liu, Beijing Academy of Agriculture and Forestry Sciences, China, personal communication). In consequence, understanding the actual epidemiological role of these strains is challenging. Similar to some “minor” PCV-2 genotypes, these strains could represent either recently emerged variants or the last descendant of previously circulating genotypes, probably like the case of PCV-2c [41,42,43]. 

The limited knowledge on PCV-3 molecular epidemiology could also justify the low Clade 2 frequency. However, the ancient origin of PCV-3 [4] and the extensive sequencing activity, especially in China, lessen the likelihood of this latter hypothesis. While Clade 1 and Clade 2 were recognized on both complete genome and ORF2 sequence analyses, an intermediate cluster could be detected at complete genome level only, as recently described by Liu et al., 2019 [44]. The reasons behind these conflicting results could be several, including differential forces acting on the Rep and Cap gene or the presence of undetected recombination events. Despite efforts were made to identify and exclude potential recombinant strains, accurate recombinant identification is challenging, especially dealing with highly homogeneous viral species like PCV-3. The actual weight of recombination in PCV-3 evolution and if its occurrence could also explain the typically low support within Clade 1 remains to be established. 

In conclusion, obtained results suggest that the current and sometimes conflicting PCV-3 classification schemes are poorly supported by sequence and biological data. With the aim of providing robust criteria that could fit an ever-increasing number of PCV-3 sequences in the future, allowing easy and automatic updates, the authors of the present work suggest the following threshold for the definition of PCV-3 genotypes: bootstrap support (or posterior probability) > 0.9, maximum genetic distance of 3% and 6% at the complete genome and ORF2 levels, and concordant results between ORF2 and the complete genome. The last criterion is dictated by practical reasons, since it would allow the genotyping of the PCV-3 strains using the ORF2 sequence only, allowing an easy classification even during routine diagnostic activity. Similarly, the congruence between NJ and more complex phylogenetic approaches could allow for fast but reliable results in everyday routine. According to this pragmatic criteria, a reference dataset has been provided (Supplementary data 1). Finally, it is suggested to formally accept a genotype only if at least five sequences are available, corresponding to approximately 1% of the analyzed PCV-3 sequences. This approach should allow a focus on more widespread genotypes and avoid the risk of defining poor quality sequences or extremely low-fitness strains as separate genotypes.

Based on these settings, we formally define only Clade 1 as PCV-3a, while Clade 2 (or other clusters that could be detected in the following years), could be recognized as a formal genotype if a larger number of strains are characterized in the future. While the relatively high number of sequences collected from countries worldwide suggests that the present data are representative of PCV-3 molecular epidemiology, other fit variants could emerge and spread, requiring a prompt classification. The nomenclature has been selected to mirror that of the well-established PCV-2 genotype classification [43].

We hope that the unified classification scheme proposed in the present study will establish a “common language” among different research groups and diagnostic laboratories. At the same time, a larger effort must be established to provide more representative and structured sampling activity and to increase the sharing of properly annotated sequences in freely available databases.

## Figures and Tables

**Table 1:** Count of sequences included in the present study classified based on the collection continent/region, country, and host. ORF2: open reading frame 2.

	Continent/Region	Country	Host						Count
			Bovine	Dog	Mouse	Unknown	Swine	Wild Boar	
Complete genome	Asia	China	4	5	4	2	243		**258**
		Japan					3		**3**
		South Korea					21		**21**
		Taiwan					2		**2**
		Thailand					2		**2**
	Central America	Mexico					1		**1**
	Europe	Denmark					2		**2**
		Germany					14		**14**
		Hungary					1		**1**
		Italy					5		**5**
		Spain					13		**13**
		Sweden					1		**1**
	Unknown	Unknown				11	1		**12**
	North America	USA				1	9		**10**
	Russia	Russia					2		**2**
	South America	Brazil					7	1	**8**
		Colombia					2		**2**
	**Total**		**4**	**5**	**4**	**14**	**329**	**1**	**357**
ORF2	Asia	China	16	18	4	6	482		**526**
		Japan					3		**3**
		South Korea					26		**26**
		Taiwan					2		**2**
		Thailand					3		**3**
	Central America	Mexico					2		**2**
	Europe	Denmark					2		**2**
		France					2		**2**
		Germany					24		**24**
		Hungary					1		**1**
		Italy					5		**5**
		Spain					14		**14**
		Sweden					1		**1**
		United Kingdom					2		**2**
	Unknown	Unknown				12	6		**18**
	North America	USA				1	9		**10**
	Russia	Russia					2		**2**
	South America	Brazil					7	1	**8**
		Colombia					2		**2**
	**Total**		**16**	**18**	**4**	**19**	**595**	**1**	**653**

**Table 2:** Results of the phylogeny–trait (i.e., region and host) association performed on both original and balanced datasets. The global statistics (AI and PS) and the feature-specific ones (MC) are reported. AI: association index; PS: parsimony score; MC: monophyletic clade size.

\		Statistic	Feature	Observed Mean	Lower Observed 95% CI	Upper Observed 95% CI	Null Mean	Lower Null 95% CI	Upper Null 95% CI	*p*-Value
Geographic location	All sequences	AI	Global	10.624	9.048	12.338	20.408	19.203	21.617	0.000
		PS		90.378	86.000	94.000	116.707	115.063	118.105	0.000
		MC	Asia	3.001	3.000	3.000	1.375	1.090	2.002	0.001
			Central America	2.000	2.000	2.000	1.025	1.000	1.102	0.001
			Europe	34.865	25.000	50.000	14.750	11.833	20.940	0.001
			North America	3.631	3.000	5.000	1.660	1.249	2.111	0.001
			Russia	2.769	2.000	3.000	1.150	1.003	1.958	0.001
			South America	1.996	2.000	2.000	1.000	1.000	1.000	0.001
			Unknown	1.973	2.000	2.000	1.000	1.000	1.000	0.001
	Balanced dataset	AI	Global	7.251	5.826	8.717	13.574	12.407	14.658	0.000
		PS		62.552	59.000	66.000	89.229	85.724	92.304	0.000
		MC	Asia	1.348	1.000	2.000	1.313	1.017	2.006	1.000
			Central America	2.000	2.000	2.000	1.118	1.000	1.812	0.026
			Europe	6.630	5.000	11.000	4.367	3.541	5.536	0.018
			North America	3.602	3.000	5.000	2.424	2.011	3.149	0.107
			Russia	3.022	2.000	4.000	1.388	1.039	2.017	0.001
			South America	2.000	2.000	2.000	1.004	1.000	1.000	0.002
			Unknown	1.873	1.000	2.000	1.003	1.000	1.000	0.001
Host	All sequences	AI	Global	5.290	4.037	6.617	9.511	8.757	10.269	0.000
		PS		41.388	39.000	44.000	53.611	52.749	53.914	0.000
		MC	Bovine	108.773	98.000	146.000	30.972	25.001	40.500	0.002
			Canine	3.612	2.000	4.000	1.107	1.013	1.339	0.001
			Mouse	6.969	6.000	7.000	1.084	1.007	1.271	0.001
			Domestic pig	1.827	1.000	2.000	1.080	1.005	1.284	0.006
			Wild boar	2.000	2.000	2.000	1.004	1.000	1.014	0.001
	Balanced dataset	AI	Global	3.082	2.345	3.900	7.543	6.736	8.316	0.000
		PS		31.276	28.000	34.000	50.186	47.890	52.021	0.000
		MC	Bovine	4.007	3.000	5.000	1.589	1.166	2.145	0.002
			Canine	17.428	17.000	19.000	4.366	3.386	6.276	0.001
			Mouse	6.662	5.000	9.000	1.472	1.110	2.062	0.001
			Domestic pig	2.310	2.000	4.000	1.481	1.110	2.083	0.160
			Wild boar	2.000	2.000	2.000	1.029	1.000	1.110	0.007

## Data Availability

All used sequences are available in Genbank and the accession number, including a suggested reference dataset, are reported in supplementary data 1.

## Supplementary Materials

The following are available online at https://www.mdpi.com/1999-4915/12/3/265/s1.
