# Supplementary figures and images for: Genotyping Porcine Circovirus 3 (PCV-3) Nowadays: Does It Make Sense?
Source: Viruses. 2020 Feb 28;12(3):265. doi: 10.3390/v12030265 (PMC7150946; doi:10.3390/v12030265)

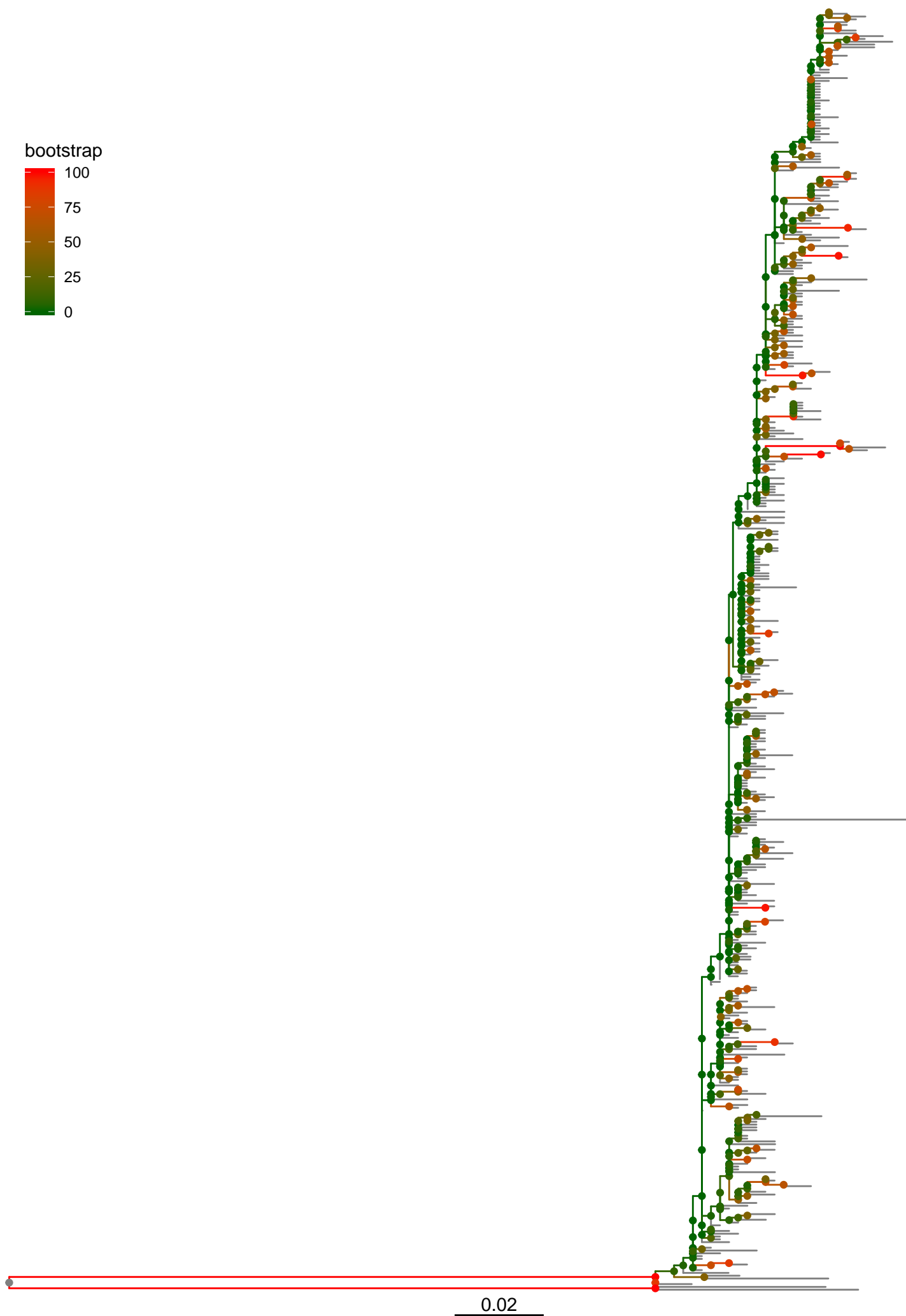

Supplement: Supplementary file 1 [file viruses-12-00265-s001.zip › Supplementary Figure 1.pdf]
